# *Wdr13* and streptozotocin-induced diabetes

**DOI:** 10.1038/s41387-018-0065-6

**Published:** 2018-10-29

**Authors:** Arun Prakash Mishra, Komala Yedella, Jyothi B. Lakshmi, Archana B. Siva

**Affiliations:** 1 CSIR-Centre for Cellular and Molecular Biology, Hyderabad, 500007 India; 2 National Cancer Institute, NIH, Frederick, MD 21702 USA

## Abstract

Type I diabetes, though contributes to only 5–10% of total diabetes cases, is a rising concern in today’s world. Our previous studies have shown that the absence of WDR13 in mouse results in pancreatic β-cell hyper-proliferation. Also, amelioration of the diabetic phenotype on introgression of *Wdr13*-null (*Wdr13*^*-/0*^) mutation in genetically diabetic mice (*Lepr*^*db/db*^) [type II diabetes] was observed. It was thus, interesting to see the role of WDR13 in streptozotocin-mediated diabetes in mice, a model for type I diabetes. *Wdr13*^*-/0*^ mice along with its wild type (*Wdr13*^*+/0*^ mice) littermates were administered streptozotocin intraperitoneally for 5 consecutive days. Blood glucose levels and body weights of these mice were monitored for subsequent 5 weeks and then they were sacrificed for physiological and histological analyses. Results showed that *Wdr13*^*-/0*^ mice exhibited higher serum insulin levels, better glucose clearance and significantly higher number of proliferating β-cells; reiterating the finding that absence of WDR13 helps in β-cell hyper-proliferation and recovery from diabetes; further underscoring WDR13 as a key target molecule for diabetes treatment/amelioration.

## Introduction

Type I diabetes, though contributes only to 5–10% of total diabetes incidence, is a rising concern for the juveniles and leads to severe consequences^1^. It is different from type II diabetes as the presence of autoantibodies, insulin dependence and insulitis are seen^2^. Susceptibility towards type I diabetes is considered to be inherited and the HLA locus alone is considered responsible for more than 50% of genetic predisposition for the same^1^. Next is the exposure to environmental triggers which alters immune system and initiates β-cell destruction. Type I diabetes has been extensively studied in mouse models^3^ and many studies have shown pancreatic regeneration ability after destruction^4–7^.

Streptozotocin (denoted as STZ from now on) administration is a readily used method to induce diabetes in rodent models and study pancreatic regeneration therein. Both multiple low-dose STZ (30–50 mg/kg body weight)^4^ and a single high dose STZ (100–200 mg/kg body weight)^7^ administration have been used to induce diabetes. High dose STZ induces necrosis and multiple low-dose causes apoptosis in β-cell lines^8^. Multiple low-dose STZ mimics type I diabetes or Insulin-Dependent Diabetes Mellitus (IDDM)^9^. β-cells take up STZ via glucose transporter GLUT2^10^ and this STZ then causes β-cell destruction via alkylation of DNA^11^.

WDR13, a protein from WD repeat protein family, was observed to be abundant in pancreas, liver, testes, and ovary^12^. *Wdr13*^*-/0*^ mice exhibit higher pancreatic islet mass and hyperinsulinemia. Absence of this protein in a type II diabetes mouse model (*Lepr*^*db/db*^) ameliorated the diabetic phenotype by increasing β-cell proliferation, resulting in higher circulating insulin^6^. In the light of above data it was interesting to study the role of WDR13 in β-cell proliferation in the type I diabetes mice model, for which we used multiple low-doses STZ-treatment to *Wdr13*^*-/0*^ mice and their wild type littermates (*Wdr13*^*+/0*^). We found that the *Wdr13*^*-/0*^ mice demonstrated better recovery from hyperglycaemia second week onwards, had better insulin levels and gained more body weight than the *Wdr13*^*+/0*^ mice.

## Materials and methods

### Animals and treatment

Mice were maintained under the guidelines of Institutional Animal Ethics Committee (IAEC) of CSIR-CCMB (Animal trial registration number-20/1999/CPCSEA dated 10/3/99). Male C57Bl/6J mice lacking WDR13 (*Wdr13*^*-/0*^) with their wild type littermates (*Wdr13*^*+/0*^) [8–10 weeks of age] were used randomly. Mice were fed on standard diet ad libitum.

Streptozotocin [Sigma, S0130] was injected intraperitoneally (dissolved in 100 mM Na-Citrate Buffer pH 4.5, 50 mg/kg body weight) in mice for 5 consecutive days. Control animals were injected with vehicle (Na-Citrate Buffer) in a similar manner. *n* = 3 for vehicle treated animals and *n* = 8 for STZ treated animals. Mice were sacrificed after 6 weeks (5 weeks after the last injection), with weekly observation of their blood glucose and body weight.

### Glucose tolerance tests (GTT)

GTT was done after 5 weeks of STZ treatment. Two different cohorts of mice were used viz—3 mice per group for control and 8 mice per group for STZ treatment, all the animals used were littermates and of 8–10 weeks of age. After 16 h of fasting, mice were injected intraperitoneally with d-glucose (1.5 g/kg body weight). Blood was sampled from mouse tail and glucose levels were analysed at every 30 min for 2 hours using Accu-Chek Active Glucometer (model number: HM100005).

### Histology

Pancreata were carefully excised off the sacrificed mice, fixed in 4% paraformaldehyde and embedded in paraffin wax. Four micrometer sections of the same were mounted on positively charged slides (Fischer scientific). Tissue histology was observed using hematoxylin-eosin staining. Islet areas were measured in pancreatic sections (three frames per section) from five different animals using Axiovision software. To study the proliferation of β-cells, immunostaining of Ki-67 (Millipore- AB9260) was performed. All immunostaining was performed according to manufacturer’s guidelines (BD Biosciences DAB substrate kit Cat.-550880). Antibody positive cells were counted manually (three frames per section) from five different animals.

### Serum collection and insulin analysis

500μl of blood drawn from the mice orbital sinus was left to clot (room temperature) for 2 hours on ice. This was then centrifuged at 10,000×*g* for 10 min and the serum was collected. ELISA kit (Millipore-EZMRI-13K) was used for analyzing serum insulin levels.

### Statistical analyses

Data are represented as mean ± SEM. We used unpaired Student’s t-test on all the data. *p < 0.05, **p < 0.01, ***p < 0.001. All studies were done without blinding. No variance analyses were done.

## Results and discussion

**Fig. 1:**
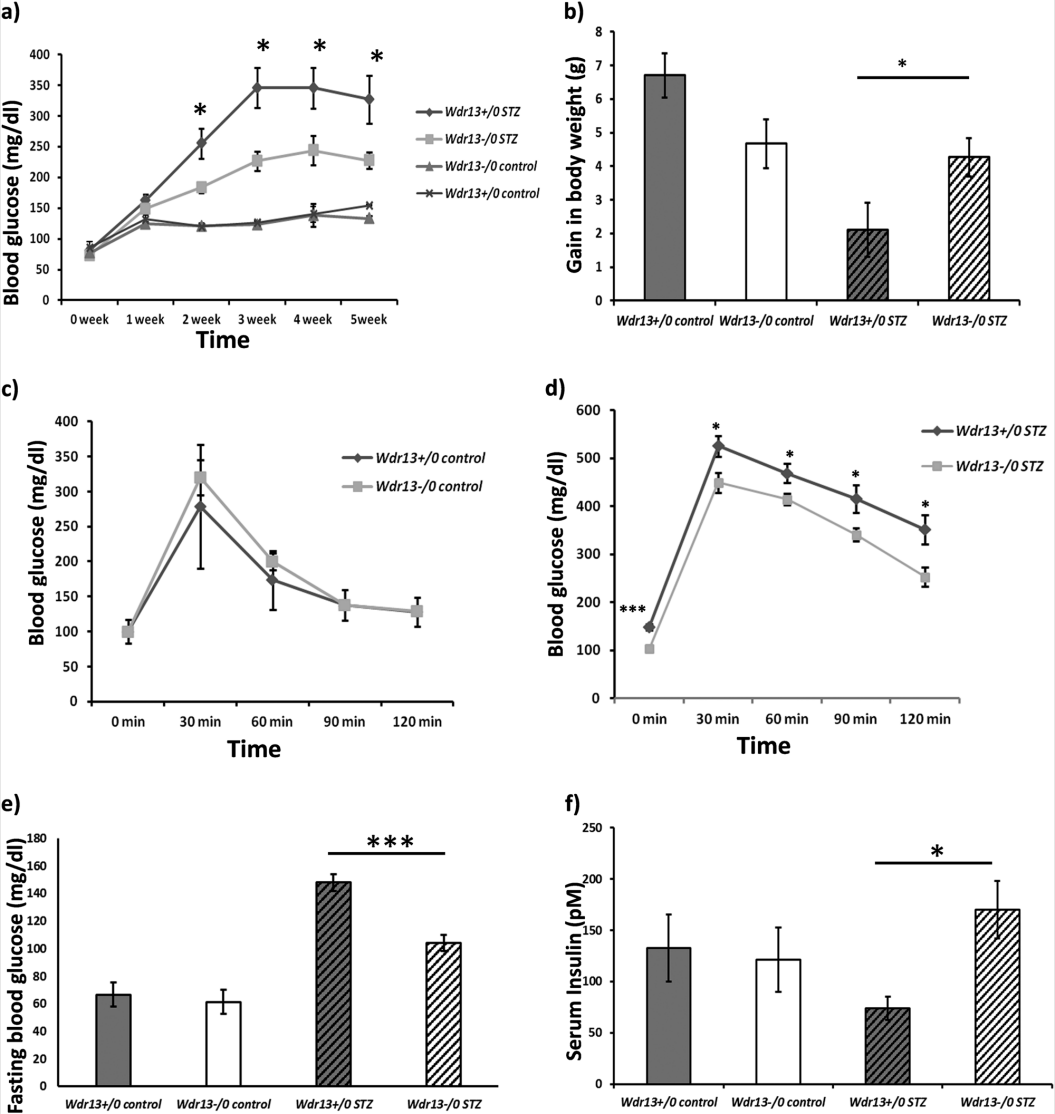
**a** Weekly blood glucose levels of vehicle and STZ treated *Wdr13*^*+/0*^ and *Wdr13*^*-/0*^ mice. **b** Gain in body weight of animals treated with vehicle and STZ. **c** Glucose tolerance test of vehicle treated animals. **d** Glucose tolerance test of STZ treated animals. **e** Fasting glucose levels of vehicle and STZ treated animals. **f** Serum insulin levels of vehicle and STZ treated *Wdr13*^*+/0*^ and *Wdr13*^*-/0*^ mice at the end of the experiment. *n* = 3 for vehicle treated animals, *n* = 8 for STZ treated animals. *p < 0.05, ***p < 0.001

Multiple low-dose STZ attacks the pancreatic β-cells, by entering into them via GLUT2 transporter and alkylating their DNA^10^. Alkylation of DNA induces apoptosis therein and a secondary trail of incidences take place via interleukins and IFNγ. This causes severe insulitis, resulting in hyperglycaemia^13^. However, pancreatic β-cells have an ability to recover from this destruction^4,5,7^. In the present study we observed a better recovery from diabetes in *Wdr13*^*-/0*^ mice as compared to *Wdr13*^*+/0*^ mice after multiple low-dose STZ administration. Weekly blood glucose monitoring displayed lower blood glucose levels in *Wdr13*^*-/0*^ mice from second week onwards, as compared to *Wdr13*^*+/0*^ mice (Fig. 1a), during the diabetes progression time of 5 weeks.

It is well known that there is a considerable amount of weight loss in diabetic condition^14,15^. This was observed in the *Wdr13*^*+/0*^ mice after multiple low-dose STZ administration. In contrast, the *Wdr13*^*-/0*^ mice performed better in maintaining their body weight (Fig. 1b) till the end of the experiment, indicating better recovery and a better health status in these animals, as compared to STZ treated *Wdr13*^*+/0*^ mice.

The STZ*-*treated *Wdr13*^*-/0*^ mice exhibited significantly better glucose clearance (GTT) as compared to the *Wdr13*^*+/0*^ mice on similar treatment (Fig. 1d). GTT conducted on vehicle administered mice showed similar rate of glucose clearance in the *Wdr13*^*-/0*^ and *Wdr13*^*+/0*^ mice (Fig. 1c). The STZ administered *Wdr13*^*-/0*^ mice also had significantly lower fasting blood glucose levels at the end of the experiment (Fig. 1e), with significantly higher serum insulin levels as compared to the *Wdr13*^*+/0*^ mice (Fig. 1f) revealing a better glucose homoeostasis.

**Fig. 2:**
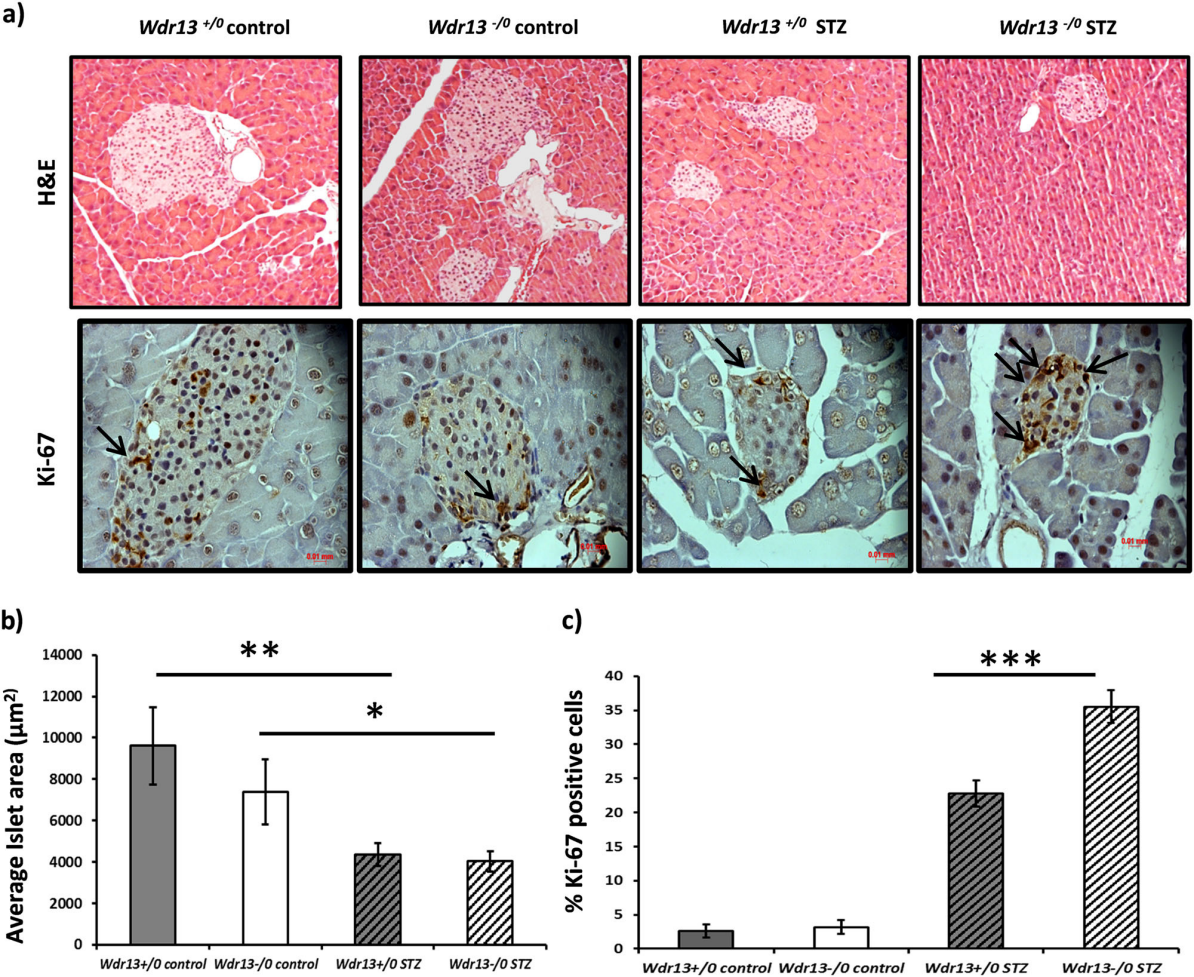
**a** Histological analyses of pancreatic sections by hematoxylin-eoisin (H&E) staining, immunostaining with Ki-67 . **b** Average islet area of vehicle and STZ treated *Wdr13*^*+/0*^ and *Wdr13*^*-/0*^ mice, determined by Axioskop software. **c** Percentage Ki-67 positive cells in STZ treated *Wdr13*^*+/0*^ and *Wdr13*^*-/0*^ mice. *p < 0.05, **p < 0.01, ***p < 0.001.

Hematoxylin-eosin staining of the pancreas showed a significant decrease in average islet size in both the groups of mice after STZ treatment; as compared to the vehicle treated animals (Fig. 2a, b). To test whether β-cell proliferation contributed to the restoration of β-cell function, Ki-67 (cell proliferation marker) immunostaining of pancreas was carried out. The results revealed more number of dividing pancreatic islet cells in STZ treated *Wdr13*^*-/0*^ mice as compared to that in *Wdr13*^*+/0*^ mice (Fig. 2a, c), confirming higher β-cell proliferation.

Pancreatic regeneration in adults is still being understood and is mainly attributed to replication of differentiated β-cells and/or neogenesis of β-cells from precursor cells^16^. However, there are important differences in the balance of these two pathways that depend upon species and age. Eventually one or both of these pathways may be manipulated for therapeutic treatment of diabetes. With regards to WDR13, we have enough evidence from this study and previous studies^6,17^ that WDR13 has a role to play in the replication of differentiated β-cells. Its role, however, in the neogenesis process has not been unequivocally addressed. One study carried out with a single high dose STZ administration (150 mg/kg body weight, dose meant to destroy all existing adult β-cells), revealed severe necrosis in the pancreatic islets of both the *Wdr13*^*+/0*^ and *Wdr13*^*-/0*^ mice and thus no recovery from diabetes, in terms of blood glucose or gain in body weight was seen, during the observation period of 30 days (Supplementary Fig. 1); suggesting a less likely role of WDR13 in the neogenesis process, even though the data available is rather preliminary.

In the previous study^6^, we showed amelioration of diabetic phenotype in absence of WDR13 in a genetically diabetic background, which makes it a type II diabetes model and in the present study it is evident that absence of WDR13 plays a protective role in type I diabetes mouse model too. We demonstrate that β-cell destruction caused due to STZ is recovered rapidly in the *Wdr13*^*-/0*^ mice as compared to *Wdr13*^*+/0*^ mice. Unlike the previous studies^6,17^ which showed β-cell hyper-proliferation in *Wdr13*^*-/0*^ mice only after 6 months of age^6,17^, here we see that happening in 2–3 months old animals after STZ insult, revealing that absence of WDR13 per se confers a proliferative advantage to β-cells, which advances the onset of phenotype in stress conditions like age, obesity, genetic background, STZ (this study), etc. To summarize, the present study reiterates that absence of WDR13 has a favourable effect on pancreatic β-cell proliferation and is likley to be a potential drug target in diabetes treatment.

## Electronic supplementary material

Supplementary figure 1
