# Supplementary material for: Wdr13 and streptozotocin-induced diabetes
Source: Nutr Diabetes. 2018 Oct 29;8:57. doi: 10.1038/s41387-018-0065-6 (PMC6204428; doi:10.1038/s41387-018-0065-6)
Supplement: Supplementary file 1 — Supplementary figure 1 [file 41387_2018_65_MOESM1_ESM.pdf]

Wdr13 and streptozotocin–induced diabetes

Arun Prakash Mishra[1,2]*, Komala Yedella[1], Jyothi B Lakshmi[1], Archana B Siva[1]
[1]CSIR- Centre for Cellular and Molecular Biology, Hyderabad -500007, India
[2]National Cancer Institute, NIH, Frederick MD-21702, USA

High dose STZ administration-

Single high dose of Streptozotocin (150mg/kg body weight) was injected intraperitoneally to *Wdr13*$^{+/0}$ and *Wdr13*$^{-/0}$ mice. Mice monitored for 30 days were severely diabetic and severe necrosis in islets was seen by H&E staining. Also they had negligible weight gain.

a)

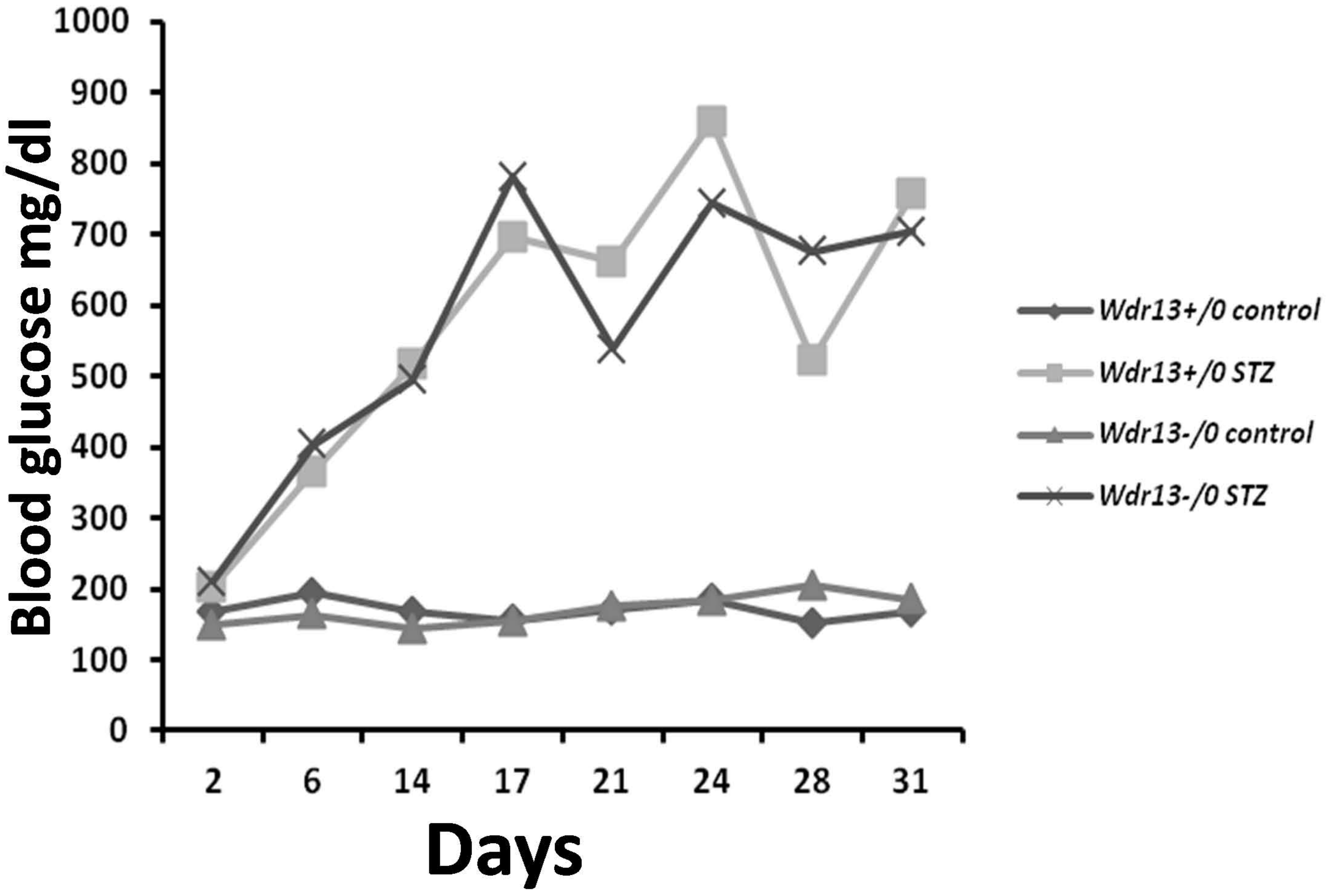

b)

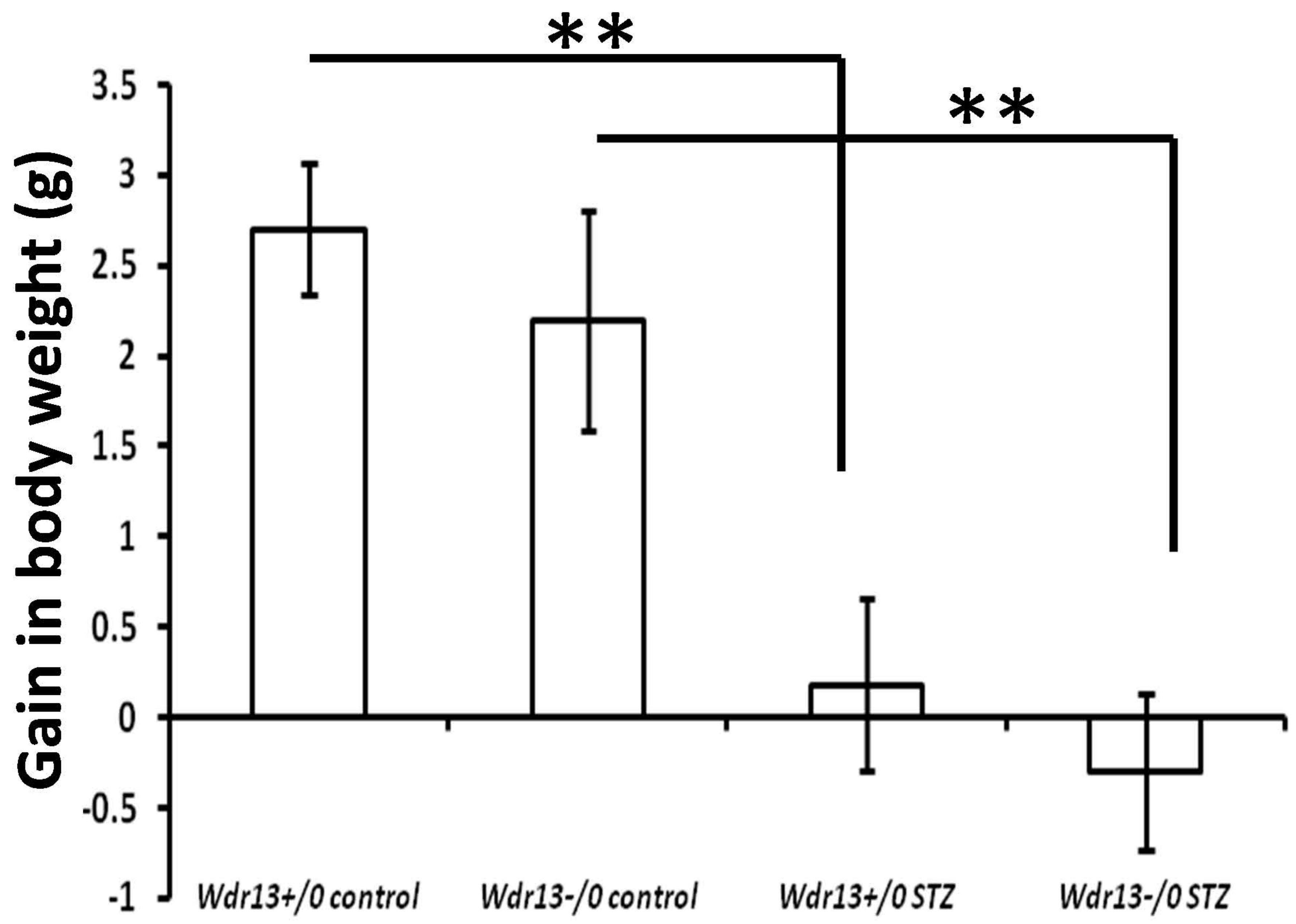

c)

Wdr13 $^{+/0}$ **control** ***Wdr13*** $^{-/0}$ **control** ***Wdr13*** $^{+/0}$ **STZ** ***Wdr13*** $^{-/0}$ **STZ**

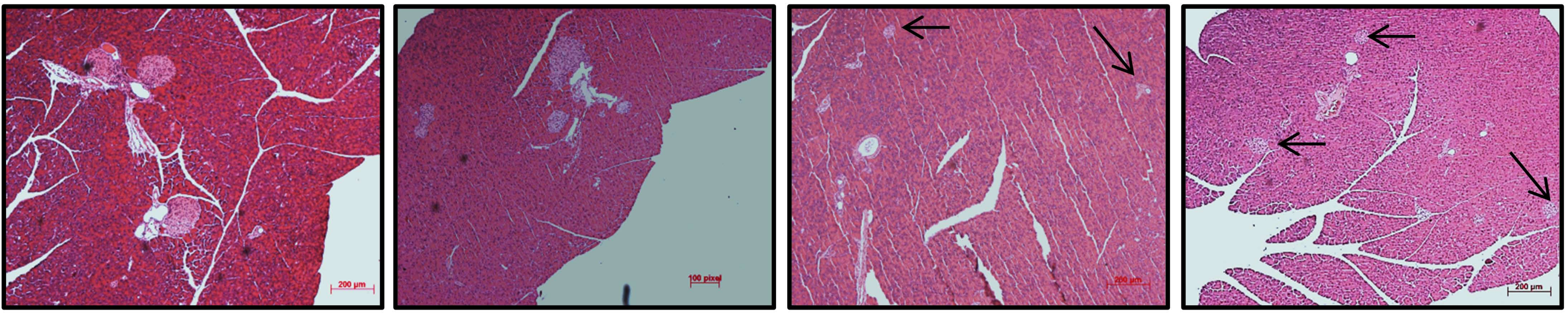

Supplementary figure 1. a) Blood glucose levels of vehicle and STZ treated *Wdr13+/0* and *Wdr13*$^{-/0}$ mice. b) Gain in body weight of animals treated with vehicle and STZ. c) Histological analyses of pancreatic sections by hematoxylin-eoisin (H&E) staining (arrows point towards islets). **p<0.01
